# Quantifying Airborne Dispersal Route of *Corynespora cassiicola* in Greenhouses

**DOI:** 10.3389/fmicb.2021.716758

**Published:** 2021-09-14

**Authors:** Qian Zhao, Yanxia Shi, Yuhong Wang, Xuewen Xie, Lei Li, Liyun Guo, Ali Chai, Baoju Li

**Affiliations:** ^1^Institute of Vegetables and Flowers, Chinese Academy of Agricultural Sciences (CAS), Beijing, China; ^2^Department of Plant Pathology, College of Plant Protection, China Agricultural University, Beijing, China; ^3^Ningbo Academy of Agricultural Sciences, Ningbo, China

**Keywords:** airborne transmission, cucumber target leaf spot, plant pathogenic fungus, air monitoring, stable production and guaranteed supply

## Abstract

Target leaf spot (TLS), caused by *Corynespora cassiicola*, is an emerging and high-incidence disease that has spread rapidly on the global scale. Aerospores released by infected plants play a significant role in the epidemiology of cucumber TLS disease; however, no data exist concerning the infectiousness and particle size of *C. cassiicola* aerospores, and the experimental evidence for the aerospores transmission was lacking. In the present study, highly effective approaches to collect and quantify aerospores were developed for exposure chamber and greenhouse studies. Quantifiable levels of *C*. *cassiicola* aerospores were detected in 27 air samples from nine naturally infested greenhouses, ranging from 198 to 5,969 spores/m^3^. The *C. cassiicola* strains isolated from air samples were infective to healthy cucumber plants. Exposure chambers were constructed to study the characteristics of *C. cassiicola* aerospores released by artificially infested cucumber plants. The particle size of *C. cassiicola* ranged predominately from 2.1 to 4.7 μm, accounting for 71.97% of the total amount. In addition, the transmission dynamics of *C. cassiicola* aerospores from donor cucumber plants to recipient cucumber plants were confirmed in exposure chambers and greenhouses. The concentration of *C. cassiicola* aerospores was positively associated with cucumber TLS disease severity. This study suggested that aerospore dispersal is an important route for the epidemiology of plant fungal disease, and these data will contribute to the development of new strategies for the effective alleviation and control of plant diseases.

## Introduction

Cucumber (*Cucumis sativus* L.) is an important vegetable crop widely cultivated worldwide. Statistically, China is the largest cucumber producer in the world, with a cultivation area of 1.26 Mha and a production of 70.34 million tons (Food and Agriculture Organization of the United Nations, 2019).^1^ In recent years, an increase in the prevalence of cucumber target leaf spot (TLS) disease has been noted in China. TLS disease is caused by the pathogenic fungus *Corynespora cassiicola* (Berk. & M.A. Curtis) C.T. Wei, which was first reported as *Helminthosporium cassiicola* in Cuba by Berkeley and Curtis (1869). The pathogen is listed as a biosafety level 1 organism due to its low bio-risk.^2^ Currently, the disease has spread globally and has been reported in East Asia, Africa, and North and South America, where it leads to substantial economic losses and threatens the livelihoods of farmers (Kwon et al., 2003; Ishii et al., 2007; Miyamoto et al., 2010). In addition, *C. cassiicola* has a wide host range, including vegetables, fruits, grains, forestry, cash crops and various ornamental plants (Dixon et al., 2009; Looi et al., 2017). The majority of commercially grown cucumber cultivars worldwide are susceptible to *C. cassiicola* (Liu et al., 2017b; Wang et al., 2018), which poses a risk to cucumber production. In the past ten years, outbreaks of TLS disease around the Bohai Sea of China resulted in 40–70% yield loss (Li et al., 2012; Liu et al., 2017b).

*C. cassiicola* is known to be transmitted by airflow, seeds, soil and rain splash (Shukla et al., 1987; Lee et al., 1990; Marcenaro and Valkonen, 2016). The pathogen is an ascomycete fungus that exists primarily as asexual spores and a vegetative mycelium in nature. The pathogen overwinters mainly on seed, soil or infected plant debris (Boosalis and Hamilton, 1957; Shukla et al., 1987), serving as a primary source of infection. Infection is caused mainly by spores that germinate to penetrate hyphae and invade healthy plants via stomata and wounds (Liu et al., 2017a). Large outbreaks of TLS disease occur by spores produced on diseased plants and transmitted by air currents (Shukla et al., 1987).

Airborne dispersal of pathogenic fungi is generally considered to be the key for the rapid spread of disease because fungal spores could potentially be transmittable over long distances (Brown and Hovmøller, 2002; Abdel-Hameed et al., 2012; Meyer et al., 2017). The first periodicals characterizing fungal capability to survive in airborne form appeared in the 1950s (Ainsworth, 1952; Thorold, 1952; Carter, 1957; Rishbeth, 1958). Subsequently, increasing knowledge has indicated that microbial aerospores are a great threat to plant health. Late blight, caused by airborne *Phytophthora infestans*, is a devastating disease of potato and is responsible for Irish potato famine, which leads to many deaths and massive emigration (Fry et al., 1993; Ristaino et al., 2001; Yuen, 2021). For destructive diseases such as rust, powdery mildew, and downy mildew, the production of huge numbers of spores, which are dispersed by wind from one susceptible host to another, leads to epidemic outbreaks and causes significant yield losses of cereals, vegetables, flowers and woodlands (Granke et al., 2014; Cao et al., 2015; Meyer et al., 2017). For the pathogenic fungus *C. cassiicola*, several studies on airborne dispersal have been conducted on rubber, cotton and sesame in the field that commonly focused on the presence of aerospores in air samples (Chee, 1988; Lee et al., 1990; Bowen et al., 2018). However, the infectiousness and particle size of aerospores remains largely vague for *C. cassiicola*, as well as for many other airborne pathogens (Coertze and Holz, 1999; Spotts and Cervantes, 2001; Scarlett et al., 2015). To date, there has been no direct evidence about the role of aerospores in the transmission of cucumber TLS disease.

A number of monitoring devices and methods have been developed to meet the demand for airborne dispersal research. The Andersen six-stage sampler has been widely used since its invention in the 1950s and is recommended as a standard bioaerosol sampler (Andersen, 1958; Brachman et al., 1964). However, their large size, long sampling time and dependence on line current (external power supply) have limited their use in remote locations. Therefore, an array of high-flow portable samplers were developed and used for sampling bioaerosols (Mehta et al., 1996; Bellin and Schillinger, 2001; He and Yao, 2011). While a high-flow portable sampler usually does not require electric outlets and is very quiet in operation, no particle size distribution data are obtained. Additionally, the high flow makes overloading more difficult to avoid than the Andersen sampler, so careful control of the sample duration is critical.

Currently, the analyses of *C. cassiicola* aerospores are generally based on conventional microscopic identification using vaselined disks of plastic film or glass slides to trap aerospores or on cultivation methods using basal media to capture aerospores (Nair and Raj, 1966; Chee, 1988; Lee et al., 1990). Actually, visual methods of identification are very labor-intensive and difficult when dealing with *C. cassiicola*, whose morphological characteristics are similar to those characteristics of many other species, which may possibly cause miscounts. In addition, the use of agar media requires several days for the fungi to grow to an identifiable stage and a certain level of expertise in fungal identification. This delay in data collection does not allow growers to take preventive action if high disease risk is predicted. In recent years, molecular techniques have increasingly been used in areas of airborne microbiology (Kennedy et al., 2000; Lee et al., 2009; Kaczmarek et al., 2019). In particular, real-time quantitative PCR (qPCR) was developed for quantitative assessment of aerospores due to its high sensitivity and specificity (An et al., 2006; Diguta et al., 2010; He and Yao, 2011). However, no method for rapid detection and quantification of *C. cassiicola* aerospores is available, nor have spore traps and DNA-based assays been deployed in China or elsewhere for this pathogen.

Airborne transmission of phytopathogenic fungi, including *C. cassiicola*, is a challenge facing a wide geographical scale, and further evidence and quantitative research on airborne dispersal of plant pathogens needs to be explicitly examined. The aim of this study was threefold: (i) to develop a highly effective approach to collect and detect *C. cassiicola* aerospores; (ii) to determine the dynamics and size distribution of *C. cassiicola* released by artificially infested cucumber plants; and (iii) to quantify airborne transmission quantities of *C. cassiicola* in exposure chambers and greenhouses. This study will present the potential of a high-volume portable sampler-qPCR combination protocol to research the epidemiology of phytopathogenic fungi in agricultural systems and provide additional evidence for airborne transmission of *C. cassiicola*. This information is beneficial for developing effective strategies to limit the spread of plant disease in China and elsewhere.

## Materials and Methods

### Fungal Strain, Inoculum Preparation and Cucumber Variety

A hygromycin-resistant (HygR) strain of *C*. *cassiicola* (*C*. *cassiicola*:HygR) was constructed and used in this study. The strain was stored at −80°C in 40% glycerol stocks and recultured on potato dextrose agar (PDA) plates (200 g of potato, 20 g of glucose and 20 g of agar per liter) containing 80 μg/mL hygromycin B (HygB) at 28°C for 5-day. The *C*. *cassiicola*:HygR strain was indistinguishable from the wild-type strain in morphological characteristics and pathogenicity (Supplementary Figures 1A,B), but was routinely grown on PDA plates containing 80 μg/mL HygB (Supplementary Figure 1C). Amplification of a 468-bp PCR product with the primers HygB-F/R (HygB-F, 5′-TGTCCTGCGGGTAAATAGC-3′; HygB-R, 5′-TTGTTGGAGCCGAAATCC-3′) confirmed that the *C*. *cassiicola*:HygR strain carried the HygB resistance gene (Supplementary Figure 1D).

When preparing the spore suspensions, 5 mm agar discs were picked and incubated for 15-day at 28°C in darkness on PDA plates containing 80 μg/mL HygB. Sterilized water with 0.05% Tween 20 was added to each agar plate, and cultures of *C. cassiicola*:HygR were washed off from the agar surfaces by gently scratching using a sterilized soft brush. The resulting suspension was filtered through four layers of sterile gauze into 50 mL conical tubes. The final *C*. *cassiicola*:HygR spore suspension was adjusted to 1 × 10^5^ spores/mL using a hemocytometer for subsequent aerosolization and inoculation experiments.

The experimental cucumber variety was “Zhongnong no. 16” (China Vegetable Seed Technology Co., Ltd., Beijing), and all seeds used in this study were first confirmed to be free of *C*. *cassiicola* by the traditional agar planting method on PDA and PCR with specific primers ga4F1 (5′-ATTGATGGGAATTGCTCTGC-3′) and ga4R1 (5′-CCTGCTCCGACTTTGTTGA-3′) (Chen et al., 2014).

### Design of Exposure Chamber

Exposure chambers made of organic glass with a size of 70 cm × 60 cm × 60 cm (length × width × height) were designed. A six-jet Collison nebulizer (BGI Inc., Waltham, United States) was connected to the air inlet located on the left-hand side of the exposure chamber. The air sampler was connected to the air outlet located on the right-hand side of the exposure chamber. A fan was installed at the top wall to ensure that the aerospores were well mixed inside the chamber. A door (20 cm × 20 cm) was fixed at the front wall with six screws, and a silicone pad was applied to ensure that the chamber was airtight. In addition, UV light was installed in the chamber for sterilization before each experiment. The air temperature was controlled at 28 ± 2°C by an air conditioner (Haier, KFRd-27N/PAA12, China), and the relative humidity was controlled under 95 ± 5% by an air humidifier (Yadu, SC-EB35B, China) through a channel preinstalled with a HEPA filter.

### Comparison of Collection Efficiency of Air Samplers

An Andersen six-stage sampler (Thermo-Andersen, Smyrna, GA, United States) and a high-volume portable sampler (HighBioTrap, Beijing Blue Tech, Inc., Beijing, China) were used to compare the collection efficiency of *C. cassiicola* aerospores in the exposure chambers based on two separate experiments.

The Andersen six-stage sampler has six stages, which have cutoff sizes of 0.65, 1.1, 2.1, 3.3, 4.7, and 7.0 μm, and different impaction velocities of 24∼1.1 m/s. The high-volume portable sampler is battery powered and has an impaction velocity of approximately 10.2 m/s and a cutoff size of ∼2 μm. A *C*. *cassiicola*:HygR spore suspension with a concentration of 1 × 10^5^ spores/mL was prepared and aerosolized into the chamber by a Collison nebulizer, which was operated at a flow rate of 12 L/min with an operating pressure of approximately 20 psi for 2 h (Sattar et al., 2017).

In the first experiment, the same sampling volumes of 500, 1,000, and 2,000 L air samples were collected by two samplers. Specifically, the sampling time was 17.5, 35, and 70 min for the Andersen sampler and 0.5, 1, and 2 min for the high-volume sampler. In the second experiment, air samples were collected for the same sampling times of 1, 2, and 5 min by two samplers. In both experiments, the Andersen sampler and the high-volume sampler were operated separately at a flow rate of 28.3 and 1,000 L/min (Chai et al., 2020). The aerospores were collected onto 90 mm diameter PDA plates containing 80 μg/mL HygB and 50 μg/mL kanamycin. After sampling, all PDA plates were cultured at 28°C for 5-day, and colony forming units (CFU) of *C*. *cassiicola*:HygR were counted. The experiments were independently repeated three times.

### Real-Time PCR Conditions and Quantification Standards

For real-time PCR (qPCR) experiments, the primer pair ga4F1/ga4R1 was used for specific amplification of *C. cassiicola*. qPCR was conducted in a 20-μL reaction volume containing 1 μL template DNA, 10 μL SuperReal PreMix Plus (Tiangen Biotech, Inc., Beijing, China), 0.4 μL of each 10 μmol/L primer, and 0.4 μL of 50 × ROX Reference Dye (Tiangen Biotech, Inc.). Amplification was performed in an Applied Biosystems 7500 Real-Time PCR System (ABI) with an initial denaturation step at 95°C for 15 min to activate the Taq polymerase, followed by 40 cycles of 95°C for 10 s and 60°C for 32 s. Cycle threshold (C_t_) values were calculated automatically by ABI7500 software.

The standard curve for the quantification of *C*. *cassiicola* was generated by analyzing a 10-fold dilution series of spore suspensions from 5 × 10^6^ to 5 spores/mL (An et al., 2006; Diguta et al., 2010). Each suspension of 1 mL was used for genomic DNA extraction by a Plant Genomic DNA Kit (Tiangen Biotech, Inc.) according to the manufacturer’s guidelines. The extractions were repeated three times for each spore concentration to create the DNA stock for the standard curve. All DNA was run in technical triplicates in 96-well reaction plates.

### Evaluation of Detection Assays for Quantifying Aerospores

Here, qPCR and plate-counting assays were compared to quantify aerospores in air samples. *C*. *cassiicola*:HygR aerospores were aerosolized into the exposure chamber from a 1 × 10^5^ spores/mL spore suspension by a Collison nebulizer as described previously and then collected onto a 90 mm diameter sterilized aluminum membrane-coated with 600 μL of mineral oil (referred to as the oil membrane) by a six-stage Andersen sampler with sampling durations of 10, 20, and 30 min.

After sampling, the oil membrane with aerospores was immediately transferred to a 50 mL centrifuge tube, washed using 3 mL of 0.05% Tween 20 suspension, and then centrifuged at 7,000 rpm for 7 min to remove the mineral oil. The pellet remaining in the centrifuge tube was resuspended in 400 μL of sterilized water. The suspensions of each sample were divided into two aliquots and assessed for the quantities of *C*. *cassiicola* by qPCR and plate-counting assay. For the plate-counting assay, a 200 μL aliquot was used for plating on agar plates and directly incubated at 28°C for 5-day; then, the CFU were counted. For qPCR, a 200 μL aliquot was used for DNA extraction, and the median C_t_ values of each triplicate were used to calculate spore numbers. The aerospore concentrations were calculated using the following equation: *C* = *N*/*V*, where *C* is the fungal concentration, *N* is the number of fungi obtained, and *V* is the total volume of air collected. The experiment was replicated three times, and each replicate was analyzed by three parallel agar plates and qPCRs.

### Sampling and Detection of *Corynespora cassiicola* Aerospores From Naturally Infested Greenhouses

To study the airborne dispersal of *C. cassiicola* in naturally infested greenhouses, aerospores were collected from nine greenhouses where cucumber TLS disease occurred naturally and one control greenhouse where cucumber grew healthy from January 2018 to February 2019. The infested greenhouses were located in Wafangdian Town, Dalian City, Liaoning Province (greenhouses I, II and III), Laoting Town, Tangshan City, Hebei Province (greenhouses IV, V and VI) and Fugou Town, Zhoukou City, Henan Province (greenhouses VII, VIII and IX). The control healthy greenhouse was located in Miyun District, Beijing City (Supplementary Table 1).

From each sampling greenhouse, two types of air samples were collected by a high-volume portable sampler that was placed 1.5 m above the ground in the middle of the greenhouse. Three independent air samples were collected from 13:00 pm to 15:00 pm daily for three continuous days. In total, 60 air samples were collected from ten greenhouses, of which 30 samples (three per greenhouse) were collected onto PDA plates, and 30 samples (three per greenhouse) were collected onto oil membranes. The disease index (DI) was examined according to the method described by Wu et al. (2018). The air temperature and relative humidity data were recorded with intervals of 5 min using a digital thermohygrometer (DL-WS 210, Gsome Technology Co., Ltd, Hangzhou, China).

For qualitative detection, the 30 samples collected onto PDA plates were assessed by fungal isolation assay. After sampling for 1 min, all PDA plates were cultured at 28°C for 3-day. The suspected *C. cassiicola* colonies were selected, transferred to a new PDA plate and cultured for 7-day. Further identification of the fungi was performed by the specific primer ga4F1/ga4R1. The pathogenicities of the isolates were also evaluated as described in our previous work (Wu et al., 2018). Briefly, cucumber seedlings (cv. Zhongnong no. 16) with two leaves were six-point inoculated with a 1 × 10^5^ spores/mL spore suspension. Healthy cucumber seedlings inoculated with sterile water served as the control. These plants were kept in a glass cabinet under 95% humidity and 28°C for 48 h and then incubated at 15°C (night)/28°C (day) in the greenhouse. Disease development was observed every day, and the DI values were examined at 7 days post inoculation (dpi). For quantitative detection, the 30 samples collected onto oil membranes were assessed by qPCR. After sampling for 5 min, all *C*. *cassiicola* aerospores were assessed by the protocol described in a previous study.

### Transmission Dynamics and Size Distribution of *Corynespora cassiicola* Aerospores in Exposure Chambers

Eighteen cucumber seedlings with two leaves were six-point inoculated with a micropipette using 10 μL of *C*. *cassiicola*:HygR spore suspension (1 × 10^5^ spores/mL) on the adaxial side of each true leaf. Healthy cucumber seedlings inoculated with sterile water served as the control. These plants were then incubated at 28 ± 2°C and 95 ± 5% RH in the exposure chamber. Aerospores were collected at 0, 7, 14, and 21 dpi. An Andersen six-stage sampler was used to collect the air samples onto 90 mm diameter PDA plates containing 80 μg/mL HygB and 50 μg/mL kanamycin. After sampling for 10 min, all PDA plates were cultured at 28°C for 5-day, and CFU of *C. cassiicola*:HygR were counted.

The size distribution of *C. cassiicola* aerospores was assessed by the Andersen sampler based on their inertia-related aerodynamic diameter (Xu et al., 2013). The DI values of cucumber TLS disease were also recorded. Pathogens were isolated from diseased cucumber leaves of *C*. *cassiicola*:HygR artificially inoculated and uninoculated; subsequently, strains in diseased leaves were detected by amplifying the ga4 and HygR genes by PCR. The experiments were independently repeated three times.

### Transmission Experiment of *Corynespora cassiicola* Aerospores in Greenhouses

The experiment was conducted from 2018 to 2019 at the Shouguang Vegetable Research and Development Centre in Xibijia Town, Shouguang City, Shangdong Province (118.94°E, 36.93°N). Three neighboring solar greenhouses (32 m × 16 m × 5.8 m, length × depth × height) were used in this case study (Supplementary Figure 2). Ventilation openings (width of 1 m) were set up on the sidewall and top of the greenhouses, which were equipped with insect proof nets to keep insects out of the greenhouses. No other cucumber plants were cultivated within approximately 500 m to ensure that the tested cucumber plants would not be infected by any pathogen from other greenhouses, and TLS disease would not spread to other greenhouses.

Cucumber seedlings were raised on October 15th and transplanted on November 8th with a plant spacing of 30 cm and row spacing of 150 cm. An inoculation center (3 m × 1.5 m, length × depth) was established at the center of the greenhouse. Cucumber seedlings planted in the inoculation center were inoculated by spraying 1 × 10^5^ spores/mL *C. cassiicola*:HygR spore suspension as donor plants, and high humidity of >95% RH was achieved by covering plastic humidity domes presprayed with sterile water. Healthy cucumber seedlings planted outside the inoculation center served as recipient plants. The plastic dome was removed when mild symptoms of TLS disease were observed on the cucumber plants in the inoculation center at 10 dpi to verify whether *C. cassiicola* spores released by the donor cucumber plants at the inoculation center could be transmitted to recipient cucumber plants outside the inoculation center. Ventilation was set at 10:00–15:00 on sunny days. A drip irrigation system was used in this study to avoid the transmission of *C. cassiicola* by water splashing. No farming operations were performed during the experiment to ensure that *C. cassiicola* could not be transmitted in other ways.

The DI values and *C. cassiicola* aerospores concentrations were investigated at different sites 0, 1.5, 3, 4.5, 6, and 7.5 m away from the inoculation center at 10, 20, 30, 40, 50, and 80 dpi. A high-volume portable sampler was used to collect air samples at 1.5 m above the ground. The sampler was operated at a sampling flow rate of 1,000 L/min, with a sampling time of 1 min for each sampling site. Air samples were collected onto a 90 mm diameter oil membrane. The concentrations of *C. cassiicola* aerospores were quantitated by qPCR. The air temperature and relative humidity data were obtained from a digital thermohygrometer. The average daily temperature and relative humidity in the greenhouse during the experiment were 14.1–37.3°C and 31.9–100% RH, respectively.

### Statistical Analysis

The concentration levels of *C. cassiicola* aerospores were recorded as the mean ± standard deviation (SD) of three independent biological replicates. Statistical analysis was conducted by either one- or two-way analysis of variance (ANOVA) using generalized linear models (GLMs) (IBM SPSS, version 24). Mean separations were performed using Tukey’s test at *P* = 0.05 and are indicated with asterisk notation.

## Results

### Comparison of Collection Efficiency of Air Samplers

**FIGURE 1:**
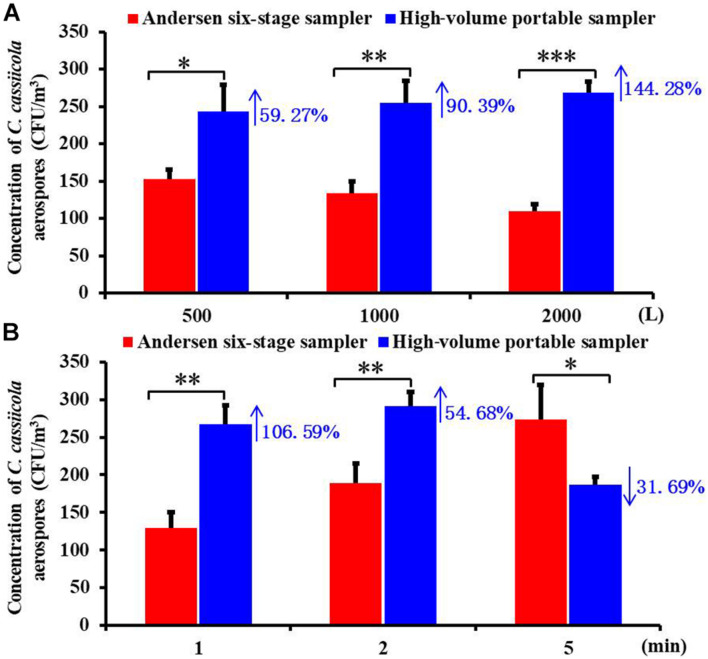
Biological collection efficiencies of Andersen six-stage sampler and high-volume portable sampler with agar plate in sampling the aerospores of aerosolized *Corynespora cassiicola* **(A)** under different sampling volumes (500, 1,000, and 2,000 L) and **(B)** under different sampling times (1, 2, and 5 min) in the exposure chamber. Data points represent averages and standard deviations of three independent sampling experiments; * indicates *P* < 0.05, ** indicates *P* < 0.01, and *** indicates *P* < 0.001.

To compare the collection efficiency of the Andersen six-stage sampler and the high-volume portable sampler, aerospores of aerosolized *C. cassiicola* were collected for the same sampling volumes and same sampling times. Significant differences between the two samplers (*P* < 0.05) were observed when sampling for 500, 1,000, and 2,000 L (Figure 1A). In general, the high-volume sampler resulted in higher *C. cassiicola* aerospore collection efficiency. Specifically, when the sampling volume was 500 L, the *C. cassiicola* aerospore concentration collected by the high-volume sampler increased 59.27% compared with that collected by the Andersen six-stage sampler, which increased to 144.28% for a sampling volume of 2,000 L. No interaction (*P* > 0.05) was found between sampling volumes and sampling device, indicating that the samplers performed consistently at all sampling volumes.

ANOVA tests indicated that the sampling device played a statistically significant role in the *C. cassiicola* aerospore concentrations regardless of the sampling times tested (*P* < 0.05) (Figure 1B). Higher concentrations of *C. cassiicola* aerospores were observed from the high-volume sampler when sampling for 1 and 2 min, which increased 106.59 and 54.68%, respectively; however, when the sampling time increased to 5 min, a decrease of 31.69% was observed. Therefore, we recommend that the high-volume portable sampler is suitable for the collection of large volume samples from greenhouse or field environments in a short time (≤2 min), and the Andersen six-stage sampler is suitable for the collection of small volume samples in the exposure chamber for a relatively long time (>5 min).

### Standard Curve for qPCR

**FIGURE 2:**
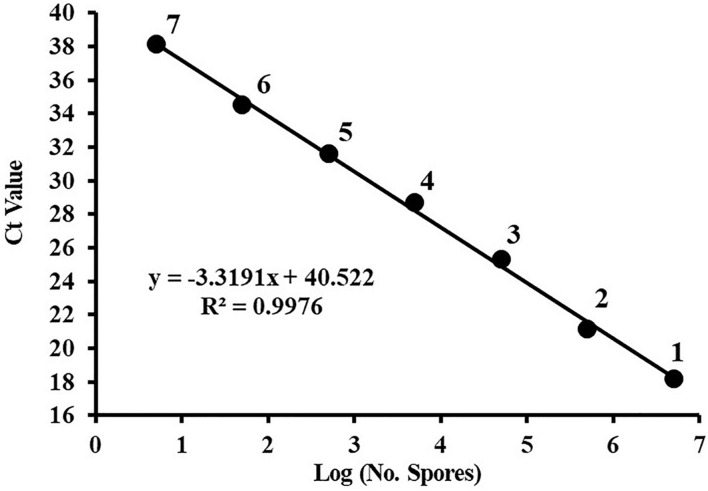
The standard curve of quantitative PCR for *Corynespora cassiicola* spores. Spores were serially diluted prior to DNA extraction, and the DNA from three replicates was pooled for each point on the standard curve. Ct, cycle threshold. Points are representative of DNA extracted from (1) 5 × 10^6^, (2) 5 × 10^5^, (3) 5 × 10^4^, (4) 5 × 10^3^, (5) 5 × 10^2^, (6) 50, and (7) 5 spores. Efficiency = 100.12%.

A dilution series of the *C*. *cassiicola*:HygR spore suspensions (5 × 10^6^ to 5 spores/mL) was prepared in sterile distilled water. The standard curve was constructed of these spore numbers against the corresponding Ct values. Plotting the log of the number of *C*. *cassiicola* spores versus the corresponding Ct values yielded a straight-line regression (y = −3.3191x + 40.522), with a correlation coefficient (*R*^2^) of 0.9976 (Figure 2). The amplification efficiency was 100.12%. Because quantification of DNA was generally unreliable (i.e., greater variance in Ct values) for DNA extracted from fewer than 5 spores, this value was considered to be the limit of detection.

### Evaluation of Detection Assays for Quantifying Aerospores

**FIGURE 3:**
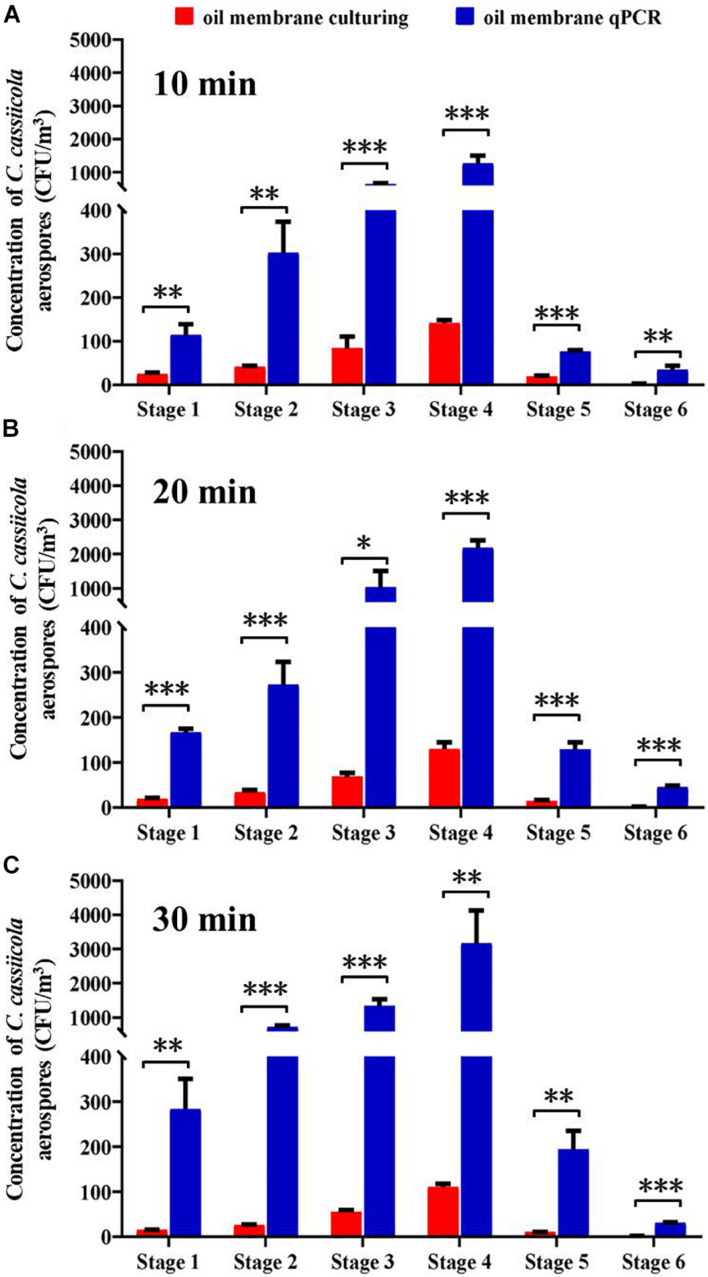
Size specific biological detection efficiencies of Andersen six-stage sampler together with oil membrane in sampling aerospores of aerosolized *Corynespora cassiicola* by culturing and qPCR methods under **(A)** 10 min, **(B)** 20 min, and **(C)** 30 min in the exposure chamber. Mineral oil (600 μL) was evenly spread onto a 90 mm diameter sterilized aluminum membrane on a Petri dish. Error bars represent the standard deviation from three independent repeats, and they are in both directions. The effects of culturing and qPCR methods on the concentrations of *C. cassiicola* aerospores were analyzed using one-way ANOVA tests. ^∗^ indicates *P* < 0.05, ^∗∗^ indicates *P* < 0.01, and ^∗∗∗^ indicates *P* < 0.001.

This study compared the counting efficiency of plate-culturing and qPCR when sampling aerosolized *C*. *cassiicola* by an Andersen six-stage sampler with an oil membrane. Overall, the *C. cassiicola* aerospore concentrations quantified using qPCR were higher than those determined using the culturing method. The use of the oil membrane coupled with qPCR resulted in approximately 4.01–39.04 times higher concentrations than the concentrations obtained using oil membrane plate-culturing. Regardless of the sampling time, the differences between the two detection methods were statistically significant (*P* < 0.05) (Figure 3).

With the increase in sampling time, the qPCR method had more times higher detection efficiency than the plate-culturing method. The *C. cassiicola* aerospore concentrations quantified using qPCR were 7.80, 14.40, and 26.55 times as high as plate-culturing when sampling for 10, 20, and 30 min, respectively (Supplementary Table 2).

### Detection of *Corynespora cassiicola* Aerospores From Naturally Infested Greenhouses

All air samples collected onto PDA plates from naturally infested greenhouses and control healthy greenhouses were analyzed by fungal isolation, morphological identification, and pathogenicity testing. A total of 69 suspected *C. cassiicola* strains were obtained from 27 air samples collected from nine naturally infested greenhouses located in different provinces (Supplementary Table 1). In the pathogenicity test, symptoms of target, pale brown, and shriveled lesions were observed on the leaves of inoculated plants at 7 dpi, and these symptoms were similar to the symptoms observed on cucumber plants under natural conditions (Supplementary Figure 3). No *C. cassiicola* strains were obtained from air samples collected from the control healthy greenhouse (Supplementary Table 1).

**FIGURE 4:**
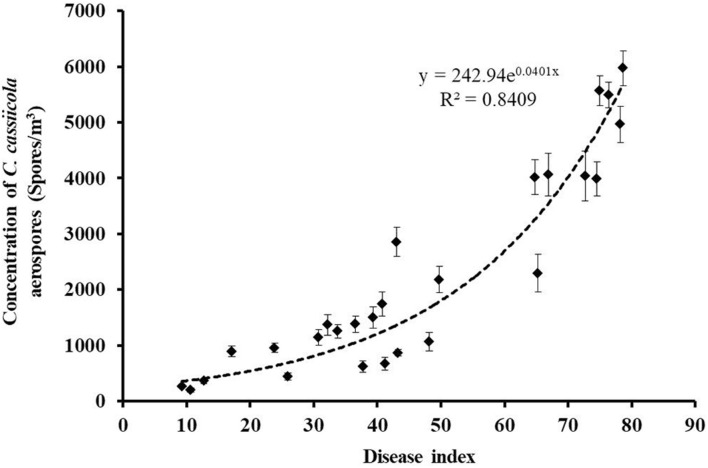
The concentrations of *Corynespora cassiicola* aerospores were plotted against the corresponding disease index values from naturally infested greenhouses. As the disease incidence increased gradually from 9.26 to 78.70, the concentration of *C. cassiicola* aerospores in greenhouses increased exponentially from 198 to 5,969 spores/m^3^. Error bars represent standard deviation (SD) from three replicates.

All air samples collected onto oil membranes from naturally infested greenhouses and control healthy greenhouses were tested for the presence of *C. cassiicola* aerospores by qPCR. *C. cassiicola* could be reliably and unequivocally quantified in all 27 air samples collected from naturally infested greenhouses, while the air samples derived from healthy greenhouses were negative for qPCR (Supplementary Table 1). The highest levels of *C. cassiicola* aerospores (5,969 spores/m^3^) were found in air samples collected from greenhouse VII located in Fugou Town, Zhoukou City, Henan Province, in 2019, with a DI of 78.70 (Sample 21). Relatively high levels of *C. cassiicola* aerospores were obtained from greenhouses I and V in Wafangdian Town, Liaoning Province, and Laoting Town, Hebei Province, with *C. cassiicola* concentrations ranging from 4,014 to 5,568 spores/m^3^, corresponding to DI values of 64.81–76.39 (Samples 1, 3, 14, and 15). The concentrations of *C. cassiicola* aerospores sampled from greenhouse II in Wafangdian Town, Liaoning Province and greenhouses IV and VI in Laoting Town, Hebei Province were fairly low; the *C. cassiicola* concentration was 1,066–1,740 spores/m^3^, corresponding to DI values of 30.79–48.15 (Samples 4–6, 10, 11, 16, and 18). The lowest level of *C. cassiicola* aerospores (198 spores/m^3^) was obtained from greenhouse III located in Wafangdian Town, Liaoning Province, in 2018, with a DI of 10.65 (Sample 7) (Supplementary Table 1). It is interesting that as the DI increased gradually from 9.26 to 78.70, the concentrations of *C. cassiicola* aerospores increased from 198 to 5,969 spores/m^3^, which indicated that diseased cucumber plants served as the main source of *C. cassiicola* aerospores. There was a direct logistic regression relationship between the concentrations of *C. cassiicola* aerospores and the DI values (Figure 4).

### Transmission Dynamics and Size Distribution of *Corynespora cassiicola* Aerospores in Exposure Chambers

**FIGURE 5:**
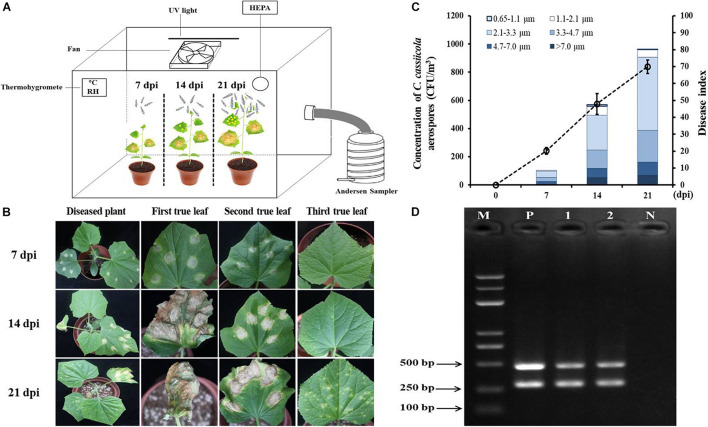
Airborne dispersal of the hygromycin-resistant (HygR) *Corynespora cassiicola* strain by artificially infested cucumber plants in the exposure chamber. **(A)** Experimental scheme of airborne dispersal analysis. The first and second true leaves of cucumber seedlings were inoculated at six points using 1 × 10^5^ spores/mL *C. cassiicola*:HygR spore suspension. *C. cassiicola*:HygR was released from inoculated leaves and transmitted to uninoculated leaves. Aerospores were collected at 0, 7, 14, and 21 dpi by an Andersen six-stage sampler. **(B)** Symptoms observed on artificially infested cucumber plants at 7, 14, and 21 dpi. **(C)** Population dynamics and size distribution of *C. cassiicola*:HygR at 7, 14, and 21 dpi. Particle size was determined by the Andersen sampler based on their inertia-related aerodynamic diameter. Error bars of the disease index represent the standard deviations of three biological replicates. **(D)** Detection of the ga4 and HygR genes in diseased leaves sampled from *C. cassiicola*:HygR-infected inoculated and uninoculated cucumber leaves by PCR. Lane M, marker; Lane 1, genomic DNA from leaves of *C. cassiicola*:HygR-infected inoculated cucumber leaves; Lane 2, genomic DNA from uninoculated cucumber leaves; Lane N, genomic DNA from leaves of control healthy cucumber plants; Lane P, positive control.

Cucumber seedlings inoculated with 1 × 10^5^ spores/mL *C. cassiicola* spore suspension were incubated in the exposure chamber (Figure 5A). As the DI values increased from 7 to 21 dpi, the concentrations of *C. cassiicola* aerospores increased gradually. At 7 dpi, typical symptoms of TLS disease appeared on the first and second true leaves of cucumber seedlings (Figure 5B). A fairly low concentration of *C. cassiicola* aerospores was obtained (102 CFU/m^3^), corresponding to a DI of 20.16 (Figure 5C). As the disease progressed, some lesions gradually enlarged and coalesced at 14 dpi. Meanwhile, symptoms of TLS disease initially appeared on the third true leaves that were not inoculated with a *C. cassiicola* spore suspension. At 21 dpi, artificially inoculated cucumber leaves were rotted and withered, and newly grown healthy leaves were seriously infected (Figure 5B). Concomitantly, the highest concentration of *C. cassiicola* aerospores (961 CFU/m^3^) was detected, corresponding to a DI value of 69.96 (Figure 5C). In addition, *C*. *cassiicola*:HygR was detected by PCR amplification of the ga4 and HygR genes from genomic DNA from both inoculated and uninoculated diseased leaves, and 290-bp ga4 and 468-bp HygR gene sequences were generated, respectively (Figure 5D). Therefore, it is reasonable to assume that *C. cassiicola* aerospores were emitted from inoculated leaves and transmitted to uninoculated leaves to cause TLS disease.

The size distribution of *C*. *cassiicola* aerospores was analyzed according to the inertia-related aerodynamic diameter of the six-stage Andersen sampler. Most of the *C. cassiicola* aerospores (approximately 71.97%) were distributed between stage 3 and stage 4 (2.1–4.7 μm), while a small number of *C. cassiicola* aerospores were detected in stage 6 (0.65–1.1 μm), corresponding to approximately 0.66%. The highest proportion of *C. cassiicola* aerospores (approximately 46.96%) was detected in stage 4 (2.1–3.3 μm). A relatively high proportion of *C. cassiicola* aerospores was detected in stage 2 (4.7–7.0 μm), at approximately 12.32%. A fairly lower proportion of *C. cassiicola* aerospores was detected in stage 1 (>7.0 μm) and stage 5 (1.1–2.1 μm), with relative proportions of 8.23 and 6.83%, respectively (Supplementary Table 3).

### Transmission Experiment of *Corynespora cassiicola* Aerospores in Greenhouses

In the inoculation center, the DI values of TLS disease increased gradually from 38.15 to 88.40, corresponding to the concentrations of *C. cassiicola* aerospores progressively increasing from 1,309 to 4,687 spores/m^3^ as the disease progressed from 10 to 80 dpi (Table 1 and Supplementary Figure 4).

**TABLE 1:** Detection of *Corynespora cassiicola* aerospores in artificially infested greenhouses.

Sampling time (dpi)	Sampling site (m)	Disease index (DI)	qPCR
			*Corynespora cassiicola* aerospore concentrations (spores/m^3^)
10 dpi	0	38.15 ± 6.16 a	1309 ± 383 a
	1.5 m	0 b	0 b
	3	0 b	0 b
	4.5	0 b	0 b
	6	0 b	0 b
	7.5	0 b	0 b
20	0	56.79 ± 4.46 a	1548 ± 507 a
	1.5	17.41 ± 5.88 b	1103 ± 380 a
	3	5.93 ± 1.11 c	236 ± 60 b
	4.5	4.07 ± 0.37 c	191 ± 21 b
	6	2.35 ± 0.77 c	178 ± 13 b
	7.5	1.23 ± 0.43 c	159 ± 29 b
30	0	64.94 ± 4.93 a	2085 ± 746 a
	1.5	29.26 ± 3.29 b	1252 ± 220 b
	3	10.49 ± 2.41 c	539 ± 169 c
	4.5	7.04 ± 2.06 c	364 ± 76 c
	6	5.31 ± 0.93 c	245 ± 36 c
	7.5	4.07 ± 0.37 c	209 ± 37 c
40	0	74.32 ± 8.13 a	3420 ± 679 a
	1.5	36.30 ± 7.52 b	1716 ± 237 b
	3	14.94 ± 2.04 c	1054 ± 316 bc
	4.5	10.99 ± 1.19 c	686 ± 131 c
	6	7.41 ± 0.37 c	422 ± 105 c
	7.5	5.80 ± 0.93 c	228 ± 48 c
50	0	75.56 ± 4.12 a	3942 ± 517 a
	1.5	34.07 ± 8.98 b	1612 ± 550 b
	3	25.31 ± 5.00 bc	1290 ± 171 bc
	4.5	18.02 ± 2.38 cd	1009 ± 228 bc
	6	15.93 ± 1.34 cd	754 ± 209 bc
	7.5	11.73 ± 2.63 d	443 ± 163 c
80	0	88.40 ± 3.80 a	4687 ± 446 a
	1.5	61.73 ± 5.56 b	3594 ± 717 b
	3	54.07 ± 2.96 bc	2074 ± 533 c
	4.5	51.36 ± 2.99 cd	1939 ± 477 c
	6	42.96 ± 3.23 de	1481 ± 140 c
	7.5	39.26 ± 3.39 e	1117 ± 328 c

*C. cassiicola aerospore concentrations are expressed as the mean ± standard deviation (SD) of data from four sampling points. Three independent experiments were performed between November 2018 and February 2019.*

*Columns labeled with different letters indicate statistically significant differences (*P* < 0.05).*

As expected, no symptoms of TLS disease were observed on plants outside the inoculation center, and no *C. cassiicola* aerospores were detected at 10 dpi before the plastic dome was removed. Ten days after the plastic dome was removed from the donor plants, symptoms of TLS disease were observed on recipient plants (Supplementary Figure 4). In general, closer to the inoculation center, more severe disease occurred, and higher levels of *C. cassiicola* aerospores were detected in the air samples. At 20 dpi, the highest levels of *C. cassiicola* aerospores (1,548 spores/m^3^) were found in air samples collected from the inoculation center, corresponding to a DI value of 56.79, and the lowest levels of *C. cassiicola* (159 spores/m^3^) were found in air samples collected 7.5 m away from the inoculation center, with a DI value of 1.23. As the disease progressed, the levels of disease severity in donor and recipient cucumber plants and the concentrations of *C. cassiicola* aerospores detected at the sampling sites increased gradually from 30 to 80 dpi. At 80 dpi, the highest levels of *C. cassiicola* aerospores (4,687 spores/m^3^) were found in air samples collected from the inoculation center, and the lowest levels of *C. cassiicola* (1,117 spores/m^3^) were found in air samples collected 7.5 m away from the inoculation area (Table 1).

## Discussion

Airborne dispersal of fungal pathogens can spread plant diseases across and even between continents (Brown and Hovmøller, 2002; Meyer et al., 2017). Some of the most striking and extreme consequences of rapid, long-distance aerial dispersal involve pathogens of crop plants, e.g., *Blumeria graminis*, *Phytophthora infestans*, *Pseudoperonospora cubensis*, and *Puccinia graminis* (Bienapfl and Balci, 2014; Granke et al., 2014; Cao et al., 2015; Simamora et al., 2015; Meyer et al., 2017; Jung et al., 2018). In the past, there have been many studies on the presence of fungal spores in air samples (Ainsworth, 1952; Thorold, 1952; Kennedy et al., 2000; Granke et al., 2014), but the study of emerging and high-incidence pathogens, e.g., *C. cassiicola* is rare. Moreover, it is important to note that the presence of fungi in the air does not mean they are infectious. In addition, most early studies on airborne dispersal of plant fungal pathogens were performed limited to field observations under uncontrolled conditions. In this study, we evaluated two sampling systems and detection methods to quantify the amount of aerosolized *C. cassiicola* aerospores. Furthermore, *C. cassiicola* aerospores were detected from nine naturally infested cucumber greenhouses in northern China, and infectivity of *C. cassiicola* strains from the air samples were verified to healthy cucumber plants. Finally, the airborne transmissibility of *C. cassiicola* was confirmed by artificially infested cucumber plants in the exposure chambers and from donor cucumber plants to recipient cucumber plants in greenhouses. The results demonstrated that high-volume portable oil membrane sampling coupled with qPCR held broad promise in monitoring airborne biological threats and provided insights into the etiological role of fungal aerospores in the transmission of plant diseases.

For airborne dispersal rated research, an effective collection method is required, and the complex nature of rapid aerial spread poses significant challenges for quantitative examination. In the present study, using the high-volume sampler resulted in higher *C. cassiicola* aerospore concentration levels than using the Andersen six-stage sampler regardless of the sampling volumes tested (500, 1,000 and 2,000 L) (Figure 1A). Possibly because the Andersen sampler is limited to low sampling flow rates, which correspondingly requires a longer sampling time (Brachman et al., 1964; Xu et al., 2013). This would be inadequate because the related problems of this sampler such as impaction stress, particle bounce, desiccation effects, and microbial embedding have not been solved (Stewart et al., 1995; Willeke et al., 1995; Mainelis and Tabayoyong, 2010). To overcome these adverse effects, high-volume sampling is increasingly being required for the monitoring of airborne microbes (Mehta et al., 1996; Bellin and Schillinger, 2001; He and Yao, 2011). However, when the sampling time increased to 5 min, significantly higher collection efficiencies were observed from the Andersen sampler than from the high-volume sampler (Figure 1B). The results were similar to the results of previous studies; the RCS High Flow (100 L/min) was shown to report higher concentration levels than the BioStage impactor (28.3 L/min) for 1 min sampling; however, when the sampling time increased to 5 min, the BioStage impactor was shown to report higher concentration levels than the RCS High Flow impactor (Zhen et al., 2009), possibly because when culture based high-volume sampling was conducted, the quantification step, involving colony counts, also presents some limitations, as plates can easily become overcrowded or overgrown, which could significantly underestimate the biological load.

In recent years, the detection of airborne fungi has been facilitated by the advancement of molecular techniques. In particular, qPCR protocols were developed for quantitative assessment of fungal counts in air samples due to the high sensitivity and specificity (Diguta et al., 2010; He and Yao, 2011; Parker et al., 2014). In the present study, using the oil membrane coupled with the qPCR method yielded significantly higher *C. cassiicola* concentrations than using the oil membrane culturing method. In general, the enhancement was shown to increase with increasing sampling time, ranging from 7.80 times at 10 min to 26.55 times at 30 min (Figure 3 and Supplementary Table 2). This finding was likely because the culturing method detected only the culturable fraction of the fungi in the air samples collected, while qPCR detected both culturable and non-culturable fungi. The non-culturable airborne fungi detected by qPCR could also present a risk because they could become culturable in a favorable environment (Wang et al., 2001; Jung et al., 2018). The increase in sampling time would affect the culturability of the fungi aerospores, thus resulting in lower fungal counts by the culturing method (Durand et al., 2002; Godish and Godish, 2007). However, the qPCR method can detect both culturable and non-culturable fungi; therefore, the sampling time would have a limited effect on airborne fungal concentrations (He and Yao, 2011). Therefore, the use of qPCR could in general improve biological exposure assessment.

The size distribution of airborne microbes is one of the main factors that impacts dispersion ability. The aerodynamic diameter of fungal spores cannot be accurately estimated solely based on the physical diameter, but additional information is needed, e.g., on the density of the spores and ambient air humidity (Reponen et al., 2001). Most previous studies on particle size distribution have focused on microbes derived from buildings, oceans and open biomass burning (Deacon et al., 2009; Lazaridis et al., 2015; Katsivela et al., 2017; Li et al., 2017; Wei et al., 2019); however, scarce information is available about the aerospore size distribution of plant fungal pathogens. In our research, the particle size of *C. cassiicola* released by cucumber plants ranged predominately from 2.1 to 4.7 μm (accounting for 71.97%), which was different from the particle size of most culturable mold fungi distributed in the 1.1–3.3 μm size range (Deacon et al., 2009; Ding et al., 2016), possibly because the average dimension of *C. cassiicola* (82.34 μm × 7.11 μm) is much larger than the average dimension of most mold fungi (3.78 μm × 3.50 μm) (unpublished data). The current lack of information on the particle size distribution of pathogens emitted from crop plants limits further study to model dispersion (Taha et al., 2007).

The emission of fungal spores to the atmosphere may play a critical role in the dispersal of plant fungal diseases. Previous studies have shown that fungal aerospore concentrations were consistently positively associated with disease severity (Lee et al., 1990; Spotts and Cervantes, 2001; Khan et al., 2009; Cao et al., 2015). The greatest numbers of *P. cubensis* airborne sporangia were detected when moderate to high disease severity (>5% symptomatic leaf area) was observed within the field, and fewer airborne sporangia were present with low disease severity (<5% symptomatic leaf area) (Granke et al., 2014). In the North Carolina study, an increase in *P. cubensis* sporangium concentrations was noted with an increasing level of disease severity until approximately 16–18% disease severity, then decreasing thereafter (Neufeld et al., 2013). In the current study, no significant decrease was noted in *C. cassiicola* aerospore concentrations with an active epidemic or as the disease progressed. A similar result was confirmed by the report that aerospores of *C. cassiicola* were at their highest concentration when the diseased crop was in full bloom or in senescent stages (Raghuram and Mallaiah, 1989). In the population dynamics experiment, an epiphytic population of *C. cassiicola* was established on cucumber leaves in the exposure chamber by inoculation with *C. cassiicola*:HygR, and the concentrations of aerospores in the chamber increased gradually as the DI values increased (Figure 5). In the transmission experiment among donor and recipient cucumber plants in greenhouses, TLS disease could be spread from the inoculation center to the non-inoculation area in 10-day, with a higher concentration of *C. cassiicola* aerospores and DI value detected in the inoculation area (Table 1 and Supplementary Figure 4). The results demonstrated that TLSs can be spread efficiently by *C. cassiicola* aerospores released by donor cucumber plants and deposited on recipient healthy cucumber plants to cause TLS disease.

Aerospore infection is thought to be the most important biofactor determining disease onset; however, the pathogenicity of airborne microbes to crop plants is largely unknown. Previous studies have shown that aerospores of *Botrytis cinerea* are pathogenic to cold-stored berries and pear fruits (Coertze and Holz, 1999; Spotts and Cervantes, 2001). Contrary evidence was apparently produced in the latest research; aerospores of *Fusarium oxysporum* f. sp. *cucumerinum* failed to establish infections of cucumber stems (Scarlett et al., 2015). In this study, *C. cassiicola* strains isolated from air samples were infective to healthy cucumber plants. Aerospores of *C. cassiicola* are deposited on the substrate surface, and infection occurs primarily through the leaf (Liu et al., 2017a), but *Fusarium* species infection occurs mainly through the root, which may explain differences in results.

## Conclusion

Our study suggests that the combination of high-volume oil membranes sampling with qPCR-based technologies is portable, timesaving, and sensitive enough for the identification and quantification of airborne microbes, especially in field applications. Moreover, our results demonstrate that airborne spread of *C. cassiicola* could explain the large outbreak of TLS disease in greenhouse. Based on culturing method, the use of a high-volume portable sampler could substantially enhance collection efficiency of *C. cassiicola* aerospores at least 54.68% increase over the Andersen sampler when sampling for less than 2 min. Compared to culturing method, the use of qPCR assays resulted in approximately 4.01–39.04 times higher concentrations. The particle size of *C. cassiicola* aerospores was distributed mainly between 2.1 and 4.7 μm. In addition, we verified that cucumber TLS disease could be transmitted by airborne dispersal both in the exposure chamber and in a controlled greenhouse, as well as the pathogenicity of *C. cassiicola* aerospores to cucumber. These findings provide insights into the potential dynamic and etiological role of fungal aerospores and contribute to the development of new strategies for the effective alleviation and control of plant diseases.

## Data Availability Statement

The original contributions presented in the study are included in the article/Supplementary Material, further inquiries can be directed to the corresponding author/s.

## Author Contributions

BL, AC, and LG designed the experiments. QZ and AC carried out the experiments. QZ, YS, YW, XX, and LL analyzed the experimental results. QZ, AC, and BL wrote the manuscript. All authors contributed to the article and approved the submitted version.

## Conflict of Interest

The authors declare that the research was conducted in the absence of any commercial or financial relationships that could be construed as a potential conflict of interest.

## Publisher’s Note

All claims expressed in this article are solely those of the authors and do not necessarily represent those of their affiliated organizations, or those of the publisher, the editors and the reviewers. Any product that may be evaluated in this article, or claim that may be made by its manufacturer, is not guaranteed or endorsed by the publisher.

## Footnotes

1
http://faostat.fao.org/

2
https://wi.knaw.nl/

## Supplementary Material

Supplementary Figure 1 Characterization of hygromycin-resistant *Corynespora cassiicola* (*C*. *cassiicola*:HygR) and *C*. *cassiicola*. **(A)** Morphological characteristics of *C*. *cassiicola*:HygR and *C*. *cassiicola* under a ×400 microscope. **(B)** Pathogenicity of *C*. *cassiicola*:HygR and *C*. *cassiicola* on cucumber seedlings. **(C)** Colony characteristics of *C*. *cassiicola*:HygR and *C*. *cassiicola* on potato dextrose agar plates containing 80 μg/mL hygromycin B. **(D)** Detection of the HygR gene in *C*. *cassiicola*:HygR and *C*. *cassiicola* by PCR with the primer pair HygB-F/R. Lane M, marker; Lane 1, plasmid pKHT-HygR; Lane 2, strain *C*. *cassiicola*:HygR; Lane 3, strain *C*. *cassiicola*; Lane N, negative control.

Supplementary Figure 2 Schematic drawing of *Corynespora cassiicola* aerospore transmission experiment among donor and recipient cucumber plants in greenhouses. The greenhouses were equipped with ventilation openings (height of 1 m) on the sidewall and the top. The inoculation center (3 m × 1.5 m) was at the center of the greenhouse. Sampling sites (marked with yellow star) were 0, 1.5, 3, 4.5, 6, and 7.5 m away from the inoculation center.

Supplementary Figure 3 Verification of the airborne dispersal of *Corynespora cassiicola* in naturally infested greenhouses. **(A1–A9)** Symptoms observed on cucumber TLS plants from greenhouse I to greenhouse IX. **(A0)** Healthy cucumber plants from the control greenhouse. **(B1–B9)** Colonies of airborne microbes collected from greenhouse I to greenhouse IX. **(B0)** Colonies of airborne microbes collected from the control greenhouse. **(C1–C9)** Colony characteristics of *C. cassiicola* strains isolated from air samples collected from greenhouse I to greenhouse IX. **(C0)** Colony characteristics of *C. cassiicola* wild-type strains. **(D1–D9)** Pathogenicity of *C. cassiicola* airborne strains from greenhouse I to greenhouse IX. **(D0)** Healthy cucumber seedlings inoculated with sterile water served as the control.

Supplementary Figure 4 Symptoms observed in the transmission experiment among donor and recipient cucumber plants in greenhouses at 10, 20, 30, 40, 50, and 80 days post inoculation (dpi). Donor cucumber plants were inoculated by spraying 1 × 10^5^ spores/mL hygromycin-resistant *Corynespora cassiicola* spore suspension in the inoculation center at 0 dpi. Sampling sites were 0, 1.5, 3, 4.5, 6, and 7.5 m away from the inoculation center.

Supplementary Table 1 Sampling and detection of *Corynespora cassiicola* aerospores in naturally infested greenhouses.

Supplementary Table 2 Quantification of *Corynespora cassiicola* aerospores by culturing and qPCR methods.

Supplementary Table 3 Size distribution of *Corynespora cassiicola* aerospores generated by cucumber after artificial inoculation.
