# Supplementary material for: Quantifying Airborne Dispersal Route of Corynespora cassiicola in Greenhouses
Source: Front Microbiol. 2021 Sep 14;12:716758. doi: 10.3389/fmicb.2021.716758 (PMC8478286; doi:10.3389/fmicb.2021.716758)
Supplement: Supplementary Figure 1 — Characterization of hygromycin-resistant Corynespora cassiicola (C. cassiicola:HygR) and C. cassiicola. (A) Morphological characteristics of C. cassiicola:HygR and C. cassiicola under a ×400 microscope. (B) Pathogenicity of C. cassiicola:HygR and C. cassiicola on cucumber seedlings. (C) Colony characteristics of C. cassiicola:HygR and C. cassiicola on potato dextrose agar plates containing 80 μg/mL hygromycin B. (D) Detection of the HygR gene in C. cassiicola:HygR and C. cassiicola by PCR with the primer pair HygB-F/R. Lane M, marker; Lane 1, plasmid pKHT-HygR; Lane 2, strain C. cassiicola:HygR; Lane 3, strain C. cassiicola; Lane N, negative control. [file Data_Sheet_1.docx]

| **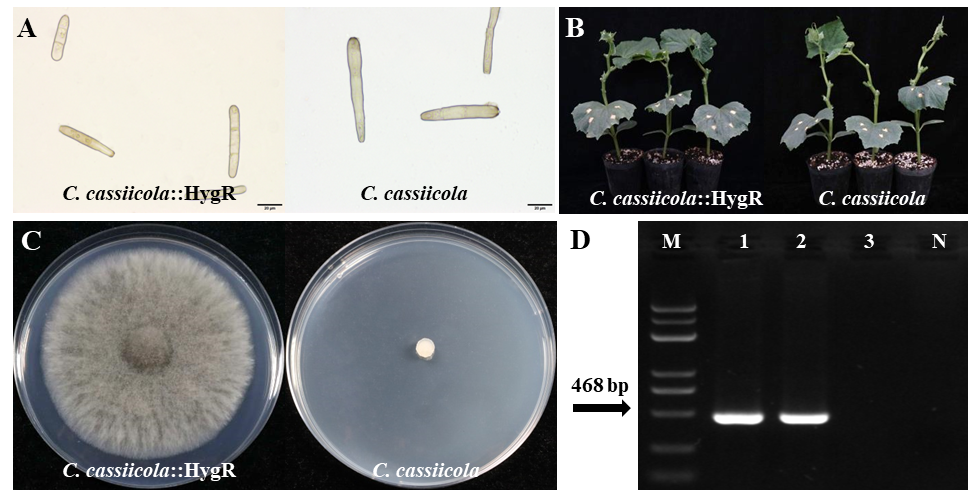** |
| --- |
| **Fig. S1** Characterization of hygromycin-resistant *Corynespora cassiicola* (*C*. *cassiicola*::HygR) and *C*. *cassiicola*. (A) Morphological characteristics of *C*. *cassiicola*::HygR and *C*. *cassiicola* under a ×400 microscope. (B) Pathogenicity of *C*. *cassiicola*::HygR and *C*. *cassiicola* on cucumber seedlings. (C) Colony characteristics of *C*. *cassiicola*::HygR and *C*. *cassiicola* on potato dextrose agar plates containing 80 μg/mL hygromycin B. (D) Detection of the HygR gene in *C*. *cassiicola*::HygR and *C*. *cassiicola* by PCR with the primer pair HygB-F/R. Lane M, marker; Lane 1, plasmid pKHT-HygR; Lane 2, strain *C. cassiicola::*HygR; Lane 3, strain *C*. *cassiicola*; Lane N, negative control. |
