# Supplementary material for: Quantifying Airborne Dispersal Route of Corynespora cassiicola in Greenhouses
Source: Front Microbiol. 2021 Sep 14;12:716758. doi: 10.3389/fmicb.2021.716758 (PMC8478286; doi:10.3389/fmicb.2021.716758)
Supplement: Supplementary Figure 2 — Schematic drawing of Corynespora cassiicola aerospore transmission experiment among donor and recipient cucumber plants in greenhouses. The greenhouses were equipped with ventilation openings (height of 1 m) on the sidewall and the top. The inoculation center (3 m × 1.5 m) was at the center of the greenhouse. Sampling sites (marked with yellow star) were 0, 1.5, 3, 4.5, 6, and 7.5 m away from the inoculation center. [file Data_Sheet_2.docx]

| 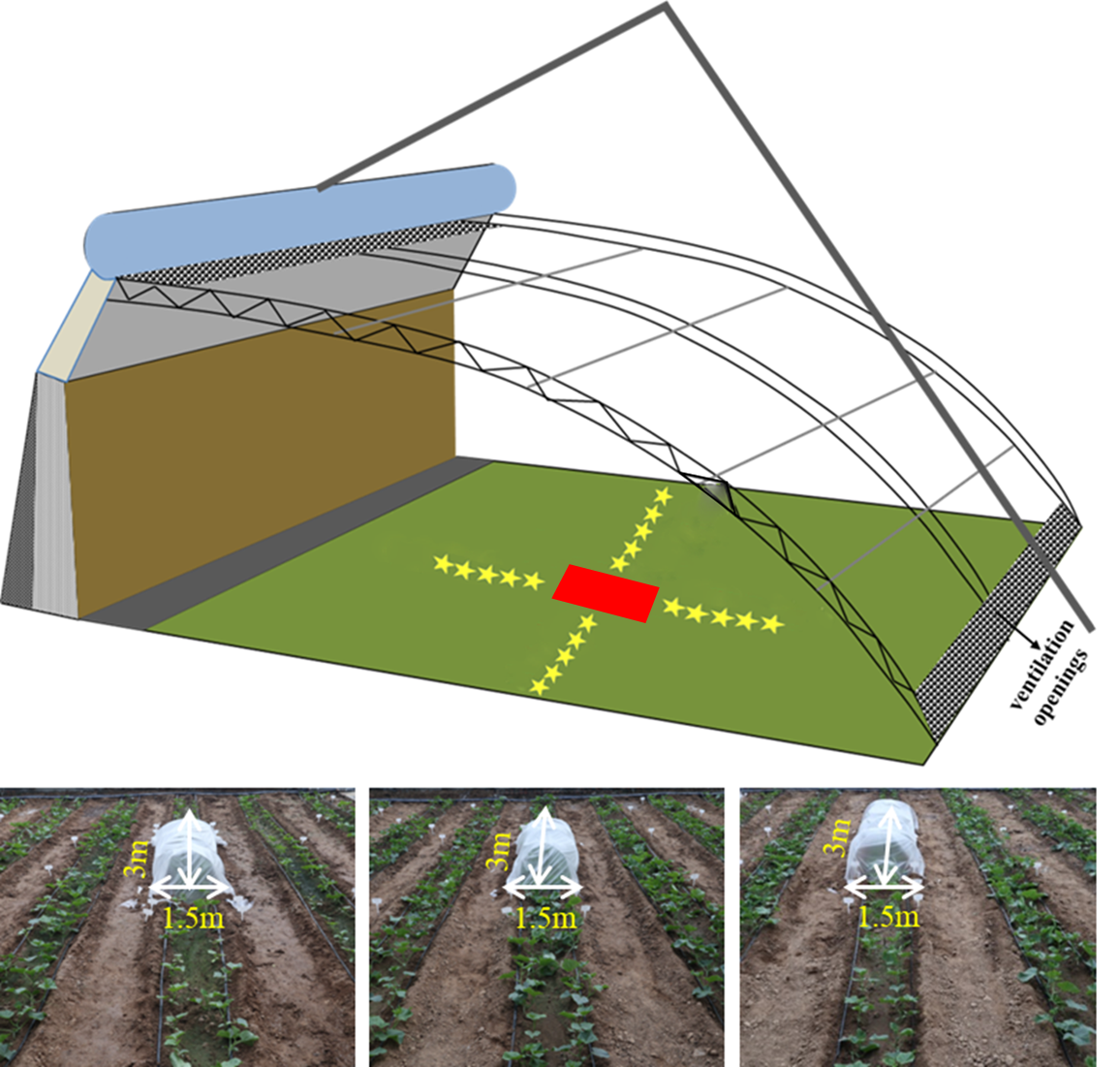 |
| --- |
| **Fig. S2** Schematic drawing of *Corynespora cassiicola* aerospore transmission experiment among donor and recipient cucumber plants in greenhouses. The greenhouses were equipped with ventilation openings (height of 1 m) on the sidewall and the top. The inoculation center (3 m × 1.5 m) was at the center of the greenhouse. Sampling sites (marked with yellow star) were 0 m, 1.5 m, 3 m, 4.5 m, 6 m and 7.5 m away from the inoculation center. |
