# Supplementary material for: Quantifying Airborne Dispersal Route of Corynespora cassiicola in Greenhouses
Source: Front Microbiol. 2021 Sep 14;12:716758. doi: 10.3389/fmicb.2021.716758 (PMC8478286; doi:10.3389/fmicb.2021.716758)
Supplement: Supplementary Figure 3 — Verification of the airborne dispersal of Corynespora cassiicola in naturally infested greenhouses. (A1–A9) Symptoms observed on cucumber TLS plants from greenhouse I to greenhouse IX. (A0) Healthy cucumber plants from the control greenhouse. (B1–B9) Colonies of airborne microbes collected from greenhouse I to greenhouse IX. (B0) Colonies of airborne microbes collected from the control greenhouse. (C1–C9) Colony characteristics of C. cassiicola strains isolated from air samples collected from greenhouse I to greenhouse IX. (C0) Colony characteristics of C. cassiicola wild-type strains. (D1–D9) Pathogenicity of C. cassiicola airborne strains from greenhouse I to greenhouse IX. (D0) Healthy cucumber seedlings inoculated with sterile water served as the control. [file Data_Sheet_3.docx]

| **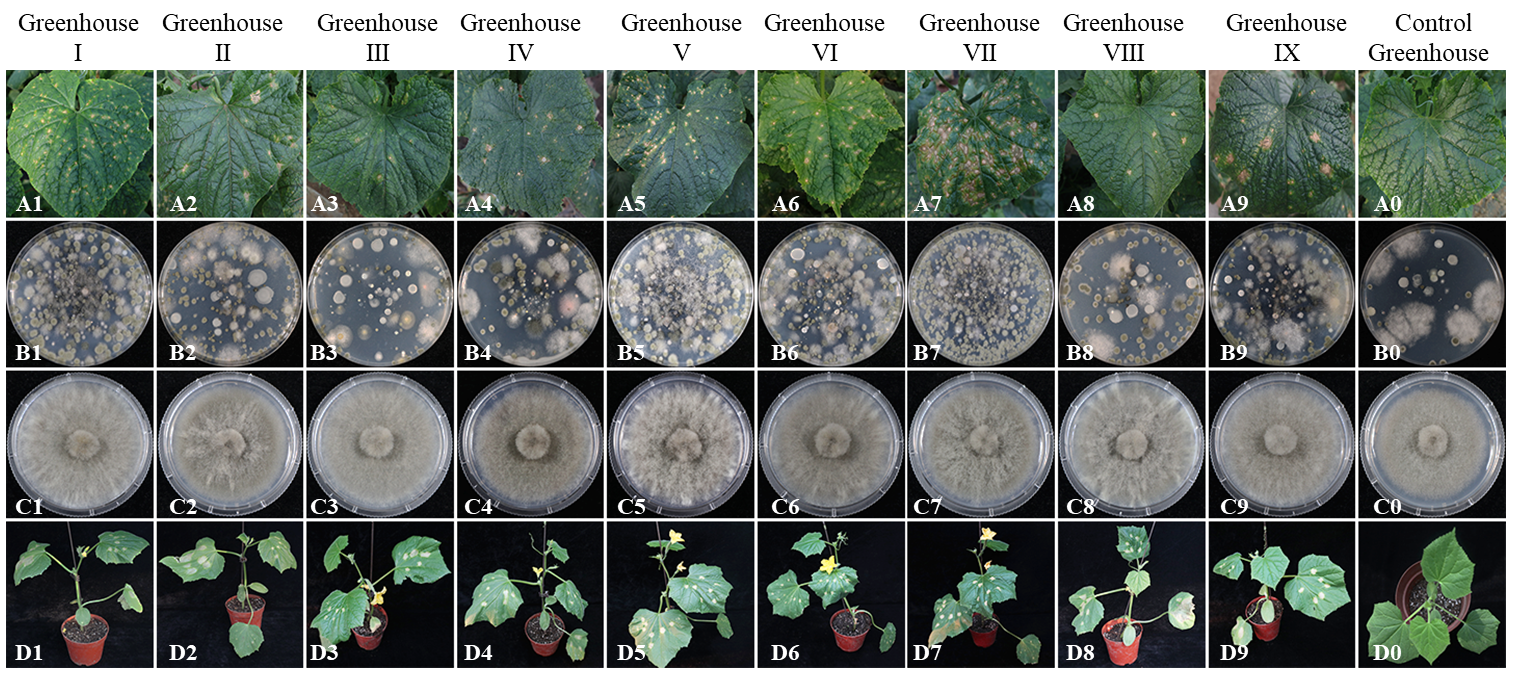** |
| --- |
| **Fig. S3** Verification of the airborne dispersal of *Corynespora cassiicola* in naturally infested greenhouses. (A1-A9): Symptoms observed on cucumber TLS plants from greenhouse Ⅰ to greenhouse Ⅸ. A0: Healthy cucumber plants from the control greenhouse. (B1-B9): Colonies of airborne microbes collected from greenhouse Ⅰ to greenhouse Ⅸ. B0: Colonies of airborne microbes collected from the control greenhouse. (C1-C9): Colony characteristics of *C. cassiicola* strains isolated from air samples collected from greenhouse Ⅰ to greenhouse Ⅸ. C0: Colony characteristics of *C. cassiicola* wild-type strains. (D1-D9): Pathogenicity of *C. cassiicola* airborne strains from greenhouse Ⅰ to greenhouse Ⅸ. D0: Healthy cucumber seedlings inoculated with sterile water served as the control. |
