# Supplementary material for: Quantifying Airborne Dispersal Route of Corynespora cassiicola in Greenhouses
Source: Front Microbiol. 2021 Sep 14;12:716758. doi: 10.3389/fmicb.2021.716758 (PMC8478286; doi:10.3389/fmicb.2021.716758)
Supplement: Supplementary Figure 4 — Symptoms observed in the transmission experiment among donor and recipient cucumber plants in greenhouses at 10, 20, 30, 40, 50, and 80 days post inoculation (dpi). Donor cucumber plants were inoculated by spraying 1 × 10^5^ spores/mL hygromycin-resistant Corynespora cassiicola spore suspension in the inoculation center at 0 dpi. Sampling sites were 0, 1.5, 3, 4.5, 6, and 7.5 m away from the inoculation center. [file Data_Sheet_4.docx]

| **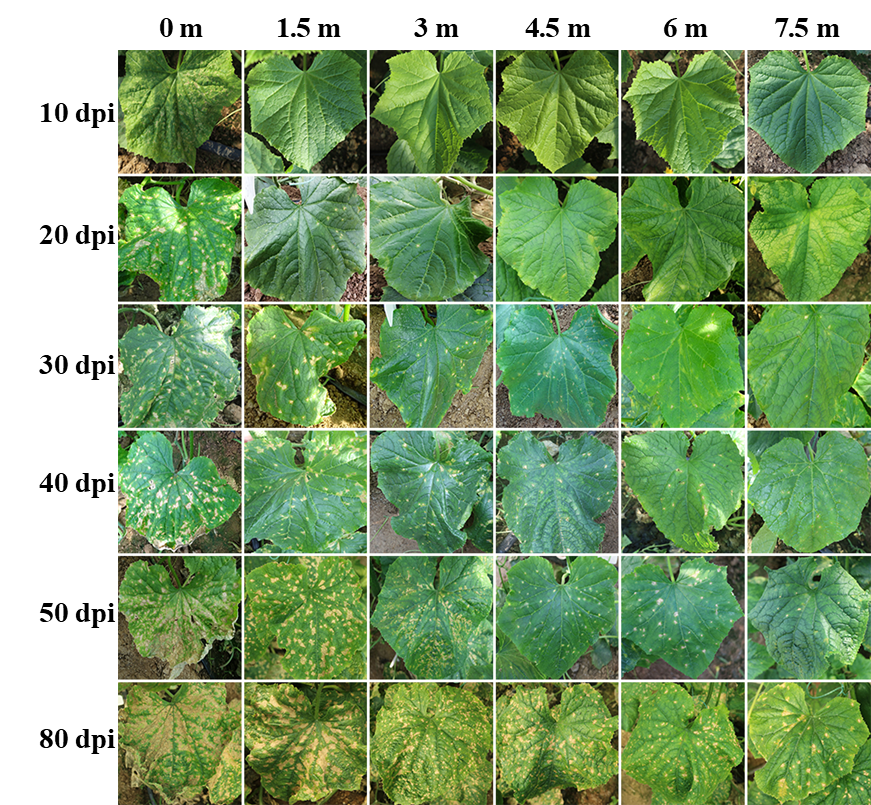** |
| --- |
| **Fig. S4** Symptoms observed in the transmission experiment among donor and recipient cucumber plants in greenhouses at 10, 20, 30, 40, 50 and 80 days post inoculation (dpi). Donor cucumber plants were inoculated by spraying 1×10^5^ spores/mL hygromycin-resistant *Corynespora cassiicola* spores suspension in the inoculation center at 0 dpi. Sampling sites were 0 m, 1.5 m, 3 m, 4.5 m, 6 m and 7.5 m away from the inoculation center. |
