# Supplementary material for: Quantifying Airborne Dispersal Route of Corynespora cassiicola in Greenhouses
Source: Front Microbiol. 2021 Sep 14;12:716758. doi: 10.3389/fmicb.2021.716758 (PMC8478286; doi:10.3389/fmicb.2021.716758)
Supplement: Supplementary Table 1 — Sampling and detection of Corynespora cassiicola aerospores in naturally infested greenhouses. [file Table_1.docx]

**Table S1** Sampling and detection of *Corynespora cassiicola* aerospores in naturally infested greenhouse.

| Location | Greenhouse No. | Sample  No. | Sampling time | Temperature and humidity | Disease index  (DI) | qPCR | Fungal isolation | |
| --- | --- | --- | --- | --- | --- | --- | --- | --- |
|  |  |  |  |  |  | *C. cassiicola* spores counts (spores/m^3^) | No. *C. cassiicola* isolated | Pathogenicity |
| Wafangdian town,  Dalian city, Liaoning Province | Ⅰ  (122.00°E, 39.53°N) | 1 | 2018.01.07 | 23.1 ~ 28.5 (26.6)°C; 50.4 ~ 64.1%RH | 76.39 | 5490 ± 236 | 4 | + |
|  |  | 2 | 2018.01.08 | 23.0 ~ 26.1 (25.3)°C; 73.5 ~ 77.4%RH | 74.54 | 3986 ± 308 | 6 | + |
|  |  | 3 | 2018.01.09 | 27.7 ~ 32.4 (31.1)°C; 45.2 ~ 52.2%RH | 75.00 | 5568 ± 267 | 5 | + |
|  | Ⅱ  (121.91°E, 39.59°N) | 4  5  6 | 2018.02.11 | 28.5 ~ 31.6 (30.5)°C; 46.7 ~ 51.7%RH | 33.80 | 1250 ± 121 | 2 | + |
|  |  |  | 2018.02.12 | 28.8 ~ 35.3 (31.3)°C; 32.0 ~ 42.6%RH | 36.57 | 1379 ± 151 | 2 | + |
|  |  |  | 2018.02.13 | 20.6 ~ 29.4 (25.6)°C; 41.9 ~ 56.0%RH | 39.35 | 1497 ± 189 | 3 | + |
|  | Ⅲ  (121.89°E, 39.64°N) | 7  8  9 | 2018.03.20 | 25.3 ~ 25.5 (25.4)°C; 19.5 ~ 20.3%RH | 10.65 | 198 ± 40 | 1 | + |
|  |  |  | 2018.03.21 | 25.3 ~ 25.6 (25.4)°C; 22.3 ~ 23.9%RH | 9.26 | 267 ± 36 | 1 | + |
|  |  |  | 2018.03.22 | 23.8 ~ 24.4 (24.1)°C; 29.1 ~ 29.8%RH | 12.73 | 359 ± 48 | 2 | + |
| Laoting town, Tangshan city, Hebei Province | Ⅳ  (118.83°E, 39.30°N) | 10  11  12 | 2018.11.22 | 26.7 ~ 35.2 (30.8)°C; 31.9 ~ 45.2%RH | 30.79 | 1142 ± 139 | 1 | + |
|  |  |  | 2018.11.23 | 27.5 ~ 36.1 (31.6)°C; 36.9 ~ 50.2%RH | 32.18 | 1369 ± 187 | 2 | + |
|  |  |  | 2018.11.24 | 16.2 ~ 20.7 (18.5)°C; 78.9 ~ 86.4%RH | 37.73 | 613 ± 103 | 3 | + |
|  | Ⅴ  (118.76°E, 39.42°N) | 13  14  15 | 2018.12.11 | 13.8 ~ 15.6 (15.0)°C; 94.0 ~ 95.9%RH | 65.28 | 2290 ± 338 | 3 | + |
|  |  |  | 2018.12.12 | 26.9 ~ 34.3 (31.1)°C; 39.0 ~ 52.9%RH | 64.81 | 4014 ± 310 | 1 | + |
|  |  |  | 2018.12.13 | 27.1 ~ 29.7 (28.9)°C; 51.3 ~ 57.8%RH | 66.90 | 4065 ± 384 | 3 | + |
|  | Ⅵ  (118.91°E, 39.51°N) | 16  17  18 | 2018.12.25 | 12.3 ~ 13.6 (13.3)°C; 79.4 ~ 97.2%RH | 48.15 | 1066 ± 162 | 2 | + |
|  |  |  | 2018.12.26 | 18.8 ~ 24.2 (21.6)°C; 46.2 ~ 62.2%RH | 49.77 | 2179 ± 232 | 1 | + |
|  |  |  | 2018.12.27 | 21.7 ~ 26.1 (24.9)°C; 60.6 ~ 69.0%RH | 40.74 | 1740 ± 214 | 3 | + |
| Fugou town, Zhoukou city, Henan Province | Ⅶ  (114.35°E, 34.02°N) | 19  20  21 | 2019.01.06 | 22.3 ~ 27.5 (25.0)°C; 39.8 ~ 63.2%RH | 72.69 | 4042 ± 446 | 4 | + |
|  |  |  | 2019.01.07 | 23.2 ~ 27.1 (25.4)°C; 63.1 ~ 72.3%RH | 78.24 | 4963 ± 328 | 5 | + |
|  |  |  | 2019.01.08 | 19.4 ~ 25.4 (22.7)°C; 48.5 ~ 57.9%RH | 78.70 | 5969 ± 318 | 1 | + |
|  | Ⅷ  (114.37°E, 34.02°N) | 22  23  24 | 2019.01.13 | 14.5 ~ 24.3 (21.6)°C; 73.4 ~ 96.6%RH | 25.93 | 434 ± 65 | 2 | + |
|  |  |  | 2019.01.14 | 23.1 ~ 26.2 (24.6)°C; 53.3 ~ 66.9%RH | 17.13 | 890 ± 92 | 3 | + |
|  |  |  | 2019.01.15 | 24.0 ~ 31.5 (28.9)°C; 48.6 ~ 62.0%RH | 23.84 | 953 ± 80 | 1 | + |
|  | Ⅸ  (114.35°E, 34.01°N) | 25  26  27 | 2019.02.21 | 23.2 ~ 29.8 (26.2)°C; 45.5 ~ 86.7%RH | 43.06 | 2853 ± 259 | 3 | + |
|  |  |  | 2019.02.22 | 15.6 ~ 21.4 (18.1)°C; 83.6 ~ 95.7%RH | 41.20 | 664 ± 114 | 2 | + |
|  |  |  | 2019.02.23 | 15.6 ~ 18.2 (16.8)°C; 94.6 ~ 97.7%RH | 43.29 | 866 ± 60 | 3 | + |
| Miyun District, Beijing city | Control  Greenhouse  (116.92°E, 40.28°N) | 28 | 2018.01.01 | 21.0 ~ 26.7 (24.7)°C; 52.1 ~ 65.4%RH | 0 | N/A | 0 | / |
|  |  | 29 | 2018.01.02 | 26.7 ~ 30.3 (28.6)°C; 40.9 ~ 57.1%RH | 0 | N/A | 0 | / |
|  |  | 30 | 2018.01.03 | 19.8 ~ 24.5 (22.4)°C; 48.4 ~ 56.0%RH | 0 | N/A | 0 | / |

The temperature per day was expressed as a range from minimum to maximum, with average value in parentheses.

*C. cassiicola* aerospore counts are expressed as the means ± standard deviations (SDs) of three replicates.

N/A (not available) means that the *C. cassiicola* aerospore counts were zero or below the detection limit.

Pathogenicity results are scored as ‘+’ for infection and disease symptoms and ‘/’ for no *C. cassiicola* strains used for the pathogenicity test.
