# Supplementary material for: Quantifying Airborne Dispersal Route of Corynespora cassiicola in Greenhouses
Source: Front Microbiol. 2021 Sep 14;12:716758. doi: 10.3389/fmicb.2021.716758 (PMC8478286; doi:10.3389/fmicb.2021.716758)
Supplement: Supplementary Table 2 — Quantification of Corynespora cassiicola aerospores by culturing and qPCR methods. [file Table_2.docx]

**Table S2** Quantification of *Corynespora cassiicola* aerospores by culturing and qPCR methods.

| Sampling time  (min) | Detection  methods | *C. cassiicola* aerospore concentrations (CFU/m^3^) | | | | | |  |
| --- | --- | --- | --- | --- | --- | --- | --- | --- |
|  |  | Stage 1  (>7.0 μm) | Stage 2  (4.7-7.0 μm) | Stage 3  (3.3-4.7 μm) | Stage 4  (2.1-3.3 μm) | Stage 5  (1.1-2.1 μm) | Stage 6  (0.65-1.1 μm) | Total |
| 10 | qPCR | 113.45 ± 25.50 | 301.71 ± 72.21 | 632.55 ± 32.83 | 1268.66 ± 238.84 | 75.61 ± 3.87 | 33.92 ± 9.22 | 2425.92 ± 257.66 |
|  | Culturing | 24.73 ± 3.53 | 41.22 ± 2.04 | 83.63 ± 26.99 | 141.34 ± 7.07 | 18.85 ± 2.04 | 1.18 ± 2.04 | 310.95 ± 35.34 |
|  | Ratio | 4.59 | 7.32 | 7.56 | 8.98 | 4.01 | 28.80 | 7.80 |
| 20 | qPCR | 166.76 ± 7.91 | 272.28 ± 50.93 | 1022.26 ± 478.84 | 2180.18 ± 222.53 | 128.90 ± 15.46 | 44.56 ± 3.94 | 3814.95 ± 630.76 |
|  | Culturing | 18.26 ± 2.70 | 33.57 ± 4.67 | 68.32 ± 8.35 | 129.56 ± 14.82 | 14.13 ± 1.77 | 1.18 ± 1.02 | 265.02 ± 31.06 |
|  | Ratio | 9.13 | 8.11 | 14.96 | 16.83 | 9.12 | 37.83 | 14.40 |
| 30 | qPCR | 238.18 ± 67.77 | 712.49 ± 53.77 | 1329.21 ± 198.10 | 3162.67 ± 967.99 | 193.56 ± 41.25 | 30.65 ± 1.26 | 5711.76 ± 1019.47 |
|  | Culturing | 14.53 ± 0.68 | 25.91 ± 1.18 | 54.97 ± 4.14 | 109.54 ± 8.24 | 9.42 ± 1.18 | 0.79 ± 0.68 | 215.16 ± 12.03 |
|  | Ratio | 19.49 | 27.50 | 24.18 | 28.87 | 20.54 | 39.04 | 26.55 |

*C. cassiicola* aerospore concentrations are expressed as the mean ± standard deviation (SD) of three replicates. The aerospores were collected using an Andersen six-stage sampler together with an oil membrane and quantified by culturing and qPCR methods under different sampling times (10, 20 and 30 min). The ratio describes the value of *C. cassiicola* aerospore concentrations between qPCR and culturing methods.
