# Supplementary material for: Quantifying Airborne Dispersal Route of Corynespora cassiicola in Greenhouses
Source: Front Microbiol. 2021 Sep 14;12:716758. doi: 10.3389/fmicb.2021.716758 (PMC8478286; doi:10.3389/fmicb.2021.716758)
Supplement: Supplementary Table 3 — Size distribution of Corynespora cassiicola aerospores generated by cucumber after artificial inoculation. [file Table_3.docx]

**Table S3** Size distribution of *Corynespora cassiicola* aerospores generated by cucumber after artificial inoculation.

| Stage | Range of | Proportion of *C. cassiicola* aerospores (%) | | | | Total | Average proportion of |
| --- | --- | --- | --- | --- | --- | --- | --- |
|  | aerodynamic diameter | 0 dpi | 7 dpi | 14 dpi | 21 dpi |  | *C. cassiicola* aerospores (%) |
| Stage 1 | ＞7.0 μm | 0.00 | 8.05 | 9.52 | 7.11 | 24.68 | 8.23 |
| Stage 2 | 4.7 – 7.0 μm | 0.00 | 16.09 | 11.18 | 9.68 | 36.95 | 12.32 |
| Stage 3 | 3.3–4.7 μm | 0.00 | 28.74 | 22.77 | 23.53 | 75.04 | 25.01 |
| Stage 4 | 2.1–3.3 μm | 0.00 | 43.68 | 43.27 | 53.92 | 140.87 | 46.96 |
| Stage 5 | 1.1–2.1 μm | 0.00 | 3.45 | 11.39 | 5.64 | 20.48 | 6.83 |
| Stage 6 | 0.65–1.1 μm | 0.00 | 0.00 | 1.86 | 0.12 | 1.98 | 0.66 |
| Total | / | 0.00 | 100 | 100 | 100 | 300 | 100 |

Cucumber seedlings were inoculated with hygromycin-resistant (HygR) *C. corynespora* strain *C. cassiicola*::HygR by dripping 1 × 10^5^ spores/mL spore suspension. Aerospores were collected at 0, 7, 14, and 21 days post inoculation (dpi). Particle size was determined by a six-stage Andersen sampler.
